# Impact of pre-existing treatment with statins on the course and outcome of tick-borne encephalitis

**DOI:** 10.1371/journal.pone.0204773

**Published:** 2018-10-04

**Authors:** Petra Bogovič, Lara Lusa, Daša Stupica, Tereza Rojko, Miša Korva, Tatjana Avšič-Županc, Klemen Strle, Gary P. Wormser, Franc Strle

**Affiliations:** 1 Department of Infectious Diseases, University Medical Center Ljubljana, Ljubljana, Slovenia; 2 Faculty of Medicine, University of Ljubljana, Ljubljana, Slovenia; 3 Institute for Biostatistics and Medical Informatics, Faculty of Medicine, University of Ljubljana, Ljubljana, Slovenia; 4 Department of Mathematics, Faculty of Mathematics, Natural Sciences and Information Technologies, University of Primorska, Koper, Slovenia; 5 Institute for Microbiology and Immunology, Faculty of Medicine, University of Ljubljana, Ljubljana, Slovenia; 6 Center for Immunology and Inflammatory Diseases, Division of Rheumatology, Allergy and Immunology, Massachusetts General Hospital, Harvard Medical School, Boston, Massachusetts, United States of America; 7 Division of Infectious Diseases, New York Medical College, Valhalla, New York, United States of America; International Centre for Genetic Engineering and Biotechnology, ITALY

## Abstract

**Objectives:**

Although statins have anti-inflammatory and potentially also antimicrobial (including antiviral) activity, their therapeutic impact on infectious diseases is controversial. In this study, we evaluated whether pre-existing statin use influenced the course and outcome of tick-borne encephalitis.

**Methods:**

To assess the influence of statin usage on the severity of acute illness and the outcome of tick-borne encephalitis, univariate and multivariable analyses were performed for 700 adult patients with tick-borne encephalitis of whom 77 (11%) were being treated with statins, and for 410 patients of whom 53 (13%) were receiving statins, respectively.

**Results:**

Multivariable analyses found no statistically significant association between statin usage and having a milder acute illness. There was also no statistically significant benefit with respect to a favorable outcome defined by the absence of post-encephalitic syndrome (ORs for a favorable outcome at 6 months was 0.96, 95% CI: 0.46–2.04, *P* = 0.926; at 12 months 0.29, 95% CI: 0.06–1.33, *P* = 0.111; at 2–7 years after acute illness 0.44, 95% CI: 0.09–2.22, *P* = 0.321), by a reduction in the frequency of six nonspecific symptoms (fatigue, myalgia/arthralgia memory disturbances, headache, concentration disturbances, irritability) occurring during the 4 week period before the last examination, or by higher SF-36 scores in any of the eight separate domains of health as well as in the physical and mental global overall component. Furthermore, there were no significant differences between patients receiving statins and those who were not in the cerebrospinal fluid or serum levels for any of the 24 cytokines/chemokines measured.

**Conclusions:**

In this observational study, we could not prove that pre-existing use of statins affected either the severity of the acute illness or the long-term outcome of tick-borne encephalitis.

## Introduction

Tick-borne encephalitis (TBE) is a central nervous system infection caused by three subtypes of TBE virus, i.e. European, Siberian and Far-Eastern. It is transmitted to humans by tick bite of the same *Ixodes* species that transmit *Borrelia burgdorferi* sensu lato, and very rarely by consumption of infected (usually goat) milk or milk products. TBE caused by the European virus subtype has a milder course and better outcome than TBE caused by the Siberian or Far-Eastern subtypes [1–3]. In the majority of patients with TBE caused by the European virus subtype, the disease has a biphasic course that begins with a nonspecific febrile illness with headache (which corresponds to viremia), followed by improvement of a few days duration, and then by the development of higher fever and signs of central nervous system involvement. However, in up to one-third of patients, the initial phase is absent or very mild. The clinical spectrum of TBE ranges from mild meningitis to severe meningoencephalitis, with or without pareses [3]. In central Europe the case fatality rate is between 0.5 and 2%, about 5% of patients are affected by permanent pareses, and at least 30% suffer from a postencephalitic syndrome (PES). There is no specific antiviral treatment for TBE [1, 3, 4]. Although potentially preventable by vaccination, infection by the TBE virus (TBEV) is responsible for more than 10,000 hospitalizations every year in endemic areas of Europe and Asia [5].

Statins are widely used drugs to lower cholesterol levels and reduce cardiovascular disease. Because these drugs have anti-inflammatory effects, as well as antimicrobial activity (including antiviral activity) [6–9], numerous studies have evaluated their impact on the outcome of a range of infectious diseases from bacterial and fungal sepsis to community acquired pneumonia [8, 10–18]. Some evidence also exists that statins may prevent neuroinflammation and blood-brain barrier dysfunction [19, 20].

The aim of this retrospective study was to evaluate whether pre-existing statin use impacted the course and outcome of TBE.

## Material and methods

### Material

Patients aged ≥ 18 years, diagnosed with TBE at the Department of Infectious Diseases, University Medical Center Ljubljana, Slovenia, in the period 2007–2012, qualified for the study. Demographic, epidemiological, laboratory and clinical data on patients were obtained prospectively, enabling a detailed analysis of the course and severity of the acute illness. In addition, the outcome of TBE was assessed at follow-up visits, 6 and 12 months after hospitalization. To assess the long-term outcome of TBE, patients diagnosed with this disease were invited to a final follow-up visit in 2014, 2–7 years after the diagnosis of TBE. At each visit, patients were asked about the presence of subjective symptoms. Symptoms that newly developed or worsened since the onset of TBE, and which had no other known medical explanation, were regarded as new or increased symptoms (NOIS) and qualified as a criterion for PES symptoms if present at ≥ 6 months after the acute illness. In addition to evaluation of subjective symptoms, during the physical examination particular attention was paid to signs of neurological involvement (tremor, ataxia, cranial and spinal nerve paralysis, etc.).

At the final visit, 2–7 years after the diagnosis of TBE, patients were asked to complete two questionnaires: 1) One that asked about the presence of six nonspecific symptoms (fatigue, arthralgias and/or myalgias, headache, concentration disorders, memory disturbances, and irritability) within the preceding 4 weeks; and 2) The SF-36 Health Survey, version 2 [SF-36v2]. Data obtained by SF-36v2 were scored according to the guidelines in Ware [21]. Scoring ranged from 0 to 100, with higher scores representing better health-related quality of life. The mean score for the general population was normalized to be 50, with a standard deviation of 10.

This study, which focused on the the impact of statins on the course and outcome of TBE in adult patients from Slovenia, was conducted in accordance with The Code of Ethics of the World Medical Association (Declaration of Helsinki) and was approved by the Medical Ethics Committee of the Ministry of Health of the Republic of Slovenia (No 152/06/13, No 178/2/13, No 37712/13). Each participant provided written informed consent. The methodological approaches used in the present study have been reported recently [4].

### Methods

Antibodies to TBEV were assessed using the Enzygnost^®^ Anti-TBE Virus (IgM, IgG) test (SiemensGmbH, Marburg, Germany) according to the manufacturer’s protocol. In addition, serologic testing for Lyme borreliosis was performed. IgM antibodies to OspC and VlsE, and IgG antibodies to VlsE borrelial antigens were determined in serum and CSF using an indirect chemiluminescence immunoassay (LIAISON, Diasorin, Italy), according to the manufacturer’s recommendations. TBEV intrathecal IgM and IgG and borrelial antibody production were calculated as described by Reiber and Peter, with values > 1.4 considered indicative of intrathecal antibody synthesis [22].

### Chemokine and cytokine determinations

The levels of 24 cytokines/chemokines associated with innate (GM-CSF, IFNα, IL-10, IL-1β, IL-6, IL-8, TNFα, CCL2, CCL3, Th1 (IFNγ, IL-12P40, IL-12P70, CXCL10, CXCL9, CCL19, Th17 (IL-17F, IL-17A, IL-22, IL-21, IL-23, IL-25, IL-27), and B cell immune response (CXCL12, CXCL13) were assessed in patient CSF and serum samples using the bead-based Luminex assays (Millipore). To minimize inter-assay variation, all measurements were performed on the same day in one complete experiment. CSF and serum for cytokine determinations were available for 13 patients who were receiving statins and 66 patients who were not treated with statins.

### Definitions

TBE was defined as a febrile illness with clinical symptoms and/or signs of meningitis or meningoencephalitis, CSF pleocytosis (> 5 × 10^6^ leukocytes/L), and demonstration of acute TBEV infection by laboratory testing (presence of serum IgM and IgG antibodies to TBEV, or demonstration of intrathecal production of IgM and/or IgG antibodies to TBEV in patients previously vaccinated against TBEV).

Patients with TBE were categorized as having: i) meningitis if they had only symptoms/signs of meningeal inflammation (fever, headache, rigidity of the neck, nausea, vomiting); ii) meningoencephalitis if they had symptoms/signs indicating brain tissue damage (impaired consciousness, concentration and cognitive function disturbances, tongue fasciculations, tremor of the extremities, focal or generalized seizures, etc.) in addition to meningitis; or iii) meningoencephalomyelitis if they also had clinical signs of alpha motor neuron injury (flaccid paresis).

### Categorization of the severity of acute illness

Patients were interpreted as having mild disease when they were diagnosed with meningitis and severe disease when they had meningoencephalitis or meningoencephalomyelitis. In the majority of patients, the severity of acute illness was also evaluated quantitatively using a standardized questionnaire, as reported previously [23]. The presence, severity, and duration of an individual symptom or sign of TBE were scored on a scale of 1–9, with the absence of a particular symptom or sign scored as zero; the severity score was defined as the sum of the individual scores [23].

### Assessment/Definition of outcome

Sequelae of TBE were defined as subjective symptoms (fatigue, headache, arthralgias and myalgias, memory and concentration disorders, emotional lability, sleep disorders, dizziness, etc.) fulfilling criteria for NOIS and as objective neurological signs (tremor, ataxia, cranial and spinal nerve pareses, etc.) present at ≥ 6 months after the acute illness. An unfavourable long-term clinical outcome (PES) was defined as the presence of ≥ 2 subjective symptoms fulfilling criteria for NOIS, and/or ≥ 1 objective neurological sign at the 6-month follow-up visit or later.

No formal testing of cognitive function was performed. However, at the final visit, 2–7 years after acute illness, the presence of six nonspecific symptoms occurring within the preceding four weeks was determined and the SF-36 Health Survey was performed.

### Statistical methods

The statistical analyses focused on the comparison of patients who were receiving statins with those who were not.

Categorical variables were summarized as frequencies and percentages, numerical variables as medians and interquartile ranges (IQRs); percentages were reported with 95% confidence intervals (CIs).

For the assessment of the association of statin use with the severity of the acute illness, patients with mild illness (meningitis) and severe illness (meningoencephalitis or meningoencephalomyelitis) were compared using univariate logistic regression. Multiple regression was used to adjust the difference between statin users and non-users for 15 other covariates, including age, male sex, underlying illnesses, vaccination against TBE, monophasic course of illness, blood leukocyte count, serum CRP level, CSF leukocyte count, CSF protein concentration, albumin quotient, IgG quotient, CSF/blood glucose concentration <0.33, level of specific TBEV serum IgG antibodies, concomitant Lyme neuroborreliosis defined by demonstration of *B*. *burgdorferi* sensu lato infection of the central nervous system by isolation of *B*. *burgdorferi* sensu lato from CSF and/or by intrathecal synthesis of *B*. *burgdorferi* sensu lato IgG or IgM antibodies, and possible concomitant Lyme borreliosis, without documentation of Lyme neuroborreliosis, as defined by the presence of *B*. *burgdorferi* sensu lato specific serum IgG antibodies as the only marker of borrelial infection. The covariates included in the multivariable model were selected using expert opinion (P.B. and F.S.) and were decided upon without knowledge of the observed outcomes. A similar approach was used to assess the association of statin usage and the severity score, using linear regression models.

Likewise, we compared the long term outcomes (defined by the presence or absence of PES at 6 months, at 12 months or at the last follow-up) of statin users and non-statin users with univariate and multivariable logistic regression models. Multivariable analysis included statins and 10 other covariates: age, male sex, underlying illnesses, severity of TBE, treatment in intensive care unit during acute disease, CSF leukocyte count, CSF protein concentration, presence of PES at a previous follow-up examination, concomitant proven Lyme neuroborreliosis, and the presence of *B*. *burgdorferi* sensu lato serum IgG antibodies as the sole manifestation of borrelial infection.

Results from regression models were summarized with unadjusted and adjusted odds ratios (ORs) for logistic regression, estimated coefficients (ECs) for linear regression, with 95% CIs, and with *P* values based on the Wald test. Statin non-users were the reference group in all the regression models; therefore, OR < 1 indicated that we estimated a lower outcome likelihood for statin users compared to non-users, while the corresponding meaning for EC value was < 0.

The differences in frequency of symptoms and SF-36 scores (obtained by questionnaire) between patients receiving statins and those who were not, were compared using Mann-Whitney rank sum test and age adjusted linear regression.

The levels of cytokines/chemokines in CSF or serum in patients who were receiving statins and in those who were not, were compared using Mann-Whitney test.

All analyses were performed using R statistical language [24]. *P* value < 0.05 was considered statistically significant.

## Results

### Severity of acute illness

A total of 717 adult patients with TBE were enrolled in the study to assess the severity of the acute illness [4]. Of the 717 patients, 17 patients were excluded from the analyses because it was not known whether they received statins. Thus, the results are based on the assessment of factors associated with the severity of the acute illness in 700 patients with TBE, 77 (11%) of whom were being treated with statins. Patients’ median age was 53 (IQR 41–63) years, 56.4% were males, and 306 (43.7%) had at least one underlying illness: most often cardiovascular disease (195, 27.9%), metabolic or endocrine disorder (99, 14.1%), or disease of the musculoskeletal system (52, 7.4%). The predominant feature of the acute illness was meningoencephalitis 437 (62.4%). Although the severity of the acute illness based on clinical parameters (meningitis—mild disease, meningoencephalitis or meningoencephalomyelitis—severe disease) was determined for all 700 patients, quantitative assessment of the severity (a numerical severity score) was available for only 460 (65.7%) of the 700 patients, i.e., for those seen in the years 2009–2012.

The patients’ basic demographic, clinical and laboratory data at the time of acute illness are shown in Table 1. The percentage of patients with mild disease (meningitis) was lower for patients who received statins (28.5%, 22/77) compared with those who were not being treated with statins (32.6%, 203/623, 95% CI for difference: -7.5 to 15.5%, OR for mild disease = 0.82, 95% CI 0.49 to 1.39; *P* = 0.48). Multivariable analysis, which adjusted the analysis for 15 other covariates, estimated a higher probability of mild disease for patients receiving statins, although these differences were also not statistically significant (OR 1.30, 95% CI: 0.69 to 2.44; *P* = 0.420).

**Table 1:** Demographic, clinical, and laboratory data on the acute illness for 700 adults with tick-borne encephalitis who were assessed for factors associated with the severity of acute illness and for 410 patients who were assessed for factors associated with the outcome of tick-borne encephalitis for whom information on treatment with statins was available.

Characteristic	Number (%, 95% CI) or median (IQR)		
	Patients assessed for factors associated with severity of acute illness (700)	Patients assessed for the outcome of TBE (410)	Patients assessed for cytokine/chemokine levels in serum and CSF (79)
Sex			
Male	395 (56.4, 52.7–60.1)	222 (54.1, 49.2–59.1)	39 (49.4; 37.9–60.9)
Female	305 (43.6, 39.9–47.3)	188 (45.9, 41.0–50.8)	40 (50.6, 39.1–62.1)
Age (years)	53 (41–63.25)	55 (42.25–63)	56 (43–62.5)
Males	52 (37–63)	53 (38–62)	57 (43–62)
Females	55 (44–64)	56 (46–64)	54.5 (44.5–63.25)
Underlying illnesses	306 (43.7, 40.0–47.5)	185 (45.1, 40.2–50.1)	38 (48.1; 36.7–59.6)
Receiving statins	77 (11, 8.8–13.6)	53 (12.9, 9.8–16.6)	13 (16.5; 9.1–26.5)
Vaccinated against TBE	26^a^ (3.7, 2.4–5.4)	16^b^ (3.9, 2.3–6.3)	0
Monophasic course of illness	273/689 (39.6, 36.0–43.4)	169/405 (41.7, 36.9–46.7)	33 (41.8; 30.8–53.4)
Clinical presentation			
Meningitis	225 (32.1, 28.7–35.7)	128 (31.2, 26.8–36.0)	33 (41.8; 30.8–53.4)
Meningoencephalitis	437 (62.4, 58.7–66.0)	265 (64.6, 59.8–69.3)	39 (49.4; 37.9–60.9)
Meningoencephalomyelitis	38 (5.4, 3.9–7.4)	17 (4.1, 2.4–6.6)	7 (8.8; 3.6–17.4)
**Severity of illness**			
According to clinical assessment			
Mild (meningitis)	225 (32.1, 28.7–35.7)	128 (31.2, 26.8–36.0)	33 (41.8; 30.8–53.4)
Severe (ME or MEM)	475 (67.9, 64.3–71.3)	282 (68.8, 64.1–73.2)	46 (58.2; 46.6–69.2)
According to severity score	12 (5–18)^c^	12 (5–18)^d^	11 (4–22.5)
Treatment in intensive care unit	58 (8.3, 6.4–10.6)	28 (6.8, 4.6–9.7)	6 (7.6; 2.8–15.8)
**Duration (days)**	7 (4–9.75)	6.5 (4.75–9)	7 (4.25–9)
**Artificial ventilation: number;**	16 (27.6, 16.7–40.9);	5 (17.9, 6.1–36.9);	2 (33.3; 4.3–77.7);
**duration (days)**	6 (3–16.25)	4 (3–7)	6 (5–7)
Duration of illness before hospitalization (days)^e^	4 (3–6)^f^	4 (3–6)^g^	4.5 (3–6)^h^
Hospitalization (days)	8 (6–11)	8 (6–10.75)	8 (5–11.5)
CSF laboratory findings			
**Leukocyte count (x 10^6^ cells/L)**	86.5 (45–155)	81.5 (40.25–139)	79 (38–139)
**Protein concentration (g/L)**	0.70 (0.54–0.92)^i^	0.70 (0.55–0.93)	0.69 (0.53–0.91)
**Elevated (> 0.45 g/L)**	612/699 (87.6, 84.9–89.9)	359 (86.7, 84.0–90.6)	14 (17.7; 10.0–27.9)
Glucose concentration (mmol/L)	3.0 (2.7–3.3)^j^	3.0 (2.7–3.4)^k^	2.9 (2.6–3.3)^l^
CSFglu/Sglu < 0.33	10/695 (1.4, 0.7–2.6)	32/408 (7.8, 5.4–10.9)	0/78 (0; 0.0–4.6)
Albumin quotient^m^	10.22 (7.85–13.88)^n^	10.12 (7.89–13.48) ^o^	10.56 (7.98–12.92)^p^
IgG quotient^m^	5.12 (3.96–6.82)^n^	5.06 (3.94–6.89) ^o^	5.18 (3.98–6.62)^p^
Concomitant Lyme neuroborreliosis^q^	21/647 (3.2, 2.0–4.9)	8/380 (2.1, 0.9–4.1)	3/76 (3.9; 0.8–11.1)
**Positive** *B*. *burgdorferi* sensu lato IgG antibodies^r^	64/641 (10.0, 7.8–12.6)	39/375 (10.4, 7.5–13.9)	7/76 (9.2; 3.8–18.1)

CI, confidence interval; IQR, interquartile range; TBE, tick-borne encephalitis; ME, meningoencephalitis; MEM, meningoencephalomyelitis; CSF, cerebrospinal fluid; CSFglu/Sglu, ratio of CSF and serum glucose concentrations.

^a^ 16 patients with complete and 10 patients with incomplete basic vaccination.

^b^ Eight patients with complete and eight patients with incomplete basic vaccination.

^c^ Data available for 460 patients.

^d^ Data available for 268 patients.

^e^ In patients with a biphasic course of illness, this figure was based on the time period from the beginning of the second (meningoencephalitic) phase until hospitalization;

^f^ Data available for 643 patients.

^g^ Data available for 378 patients.

^h^ Data available for 76 patients.

^i^ Data available for 699 patients.

^j^ Data available for 695 patients.

^k^ Data available for 408 patients.

^l^ Data available for 78 patients.

^m^ Albumin (IgG) quotient between CSF and serum albumin (IgG) concentrations; albumin quotient was interpreted to be elevated when >0.0074, IgG quotient when >0.0035.

^n^ Data available for 525 patients.

^o^ Data available for 306 patients.

^p^ Data available for 75 patients.

^q^ Demonstration of *B*. *burgdorferi* sensu lato infection of the central nervous system by isolation of *B*. *burgdorferi* sensu lato from CSF or intrathecal synthesis of *B*. *burgdorferi* sensu lato specific IgG or IgM antibodies.

^r^ The only marker of borrelial infection.

When the severity of acute illness was assessed based on the severity score (a lower score indicates a less severe illness), patients receiving statins had on average a 3.1 point higher score than non-statin treated patients. Based on univariate linear regression with severity score as outcome, the EC for receiving statins was 3.10 (95% CI: 0.56 to 5.6; *P* = 0.017). However, the estimated difference was close to 0 when the analysis was adjusted for the other covariates using multivariable analysis (EC = -0.15, 95% CI: -2.83 to 2.53; *P* = 0.914).

### Outcome of tick-borne encephalitis

Four-hundred-twenty TBE patients were assessed for severity of the acute illness at the initial visit and were also evaluated at 2–7 years thereafter to assess the long-term outcome of TBE. Information on treatment with statins was available for 410 of these 420 patients.

Demographic, clinical and laboratory findings at the time of acute illness for these patients are reported in Table 1. The percentage of patients without PES was very similar for statin users and non-statin users at all three time points: 6 months (59% and 58.5%), 12 months (62.5% vs 69%) and at the final visit (62% vs 68%), respectively. OR for the absence of PES from univariate logistic regression was 1.02 (95% CI: 0.53–1.96, *P* = 0.941) at 6 months, while the outcome was slightly worse at 12 months (OR = 0.76, 95% CI: 0.34–1.67, *P* = 0.483) and at the final visit, 2–7 years after acute illness (OR = 0.76, 95% CI: 0.42–1.39, *P* = 0.378), but the differences were not statistically significant.

When the analysis of statin use on long-term outcome was adjusted for 10 other covariates, there were no statistically significantly differences associated with statin use and a favorable outcome, as defined by the absence of PES. The corresponding OR for a favorable outcome at 6 months was 0.96 (95% CI: 0.46–2.04, *P* = 0.926), at 12 months it was 0.29 (95% CI: 0.06–1.33, *P* = 0.111), and at 2–7 years after acute illness it was 0.44 (95% CI: 0.09–2.22, *P* = 0.321).

Patients receiving statins were more likely to report a greater number of symptoms (out of the 6 symptoms assessed) within 4 weeks before the final evaluation, however only fatigue, myalgia/arthralgia and memory disturbances were statistically significantly higher than in those not treated with statins (*P* = 0.0180, *P* < 0.0001, and *P* = 0.0003, respectively). No significant differences were observed for headache, concentration disturbances or irritability.

Comparison of SF-36 findings revealed that of the eight separate domains of health, patients receiving statins scored statistically significantly lower in physical functioning (*P* = 0.0004) and general health perceptions (*P* = 0.0342) than patients not receiving statins. Differences in the other six domains (social functioning, body pain, limitations due to emotional problems and due to physical health problems, mental health and vitality) were not significant. Patients receiving statins also had lower scores in the two global overall components, although the difference was statistically significant only for the physical (*P* = 0.005) but not the mental component (*P* = 0.4475).

Patients receiving statins were older than those who were not treated with statins (median 68 (62–74) years versus 57 (IQR 45–66) years; *P* < 0.0001) and more often had an underlying illness (47/53, 88.7% versus 138/357, 38.7%; *P* < 0.0001). When adjusted for age, the estimated differences in the frequency of six nonspecific symptoms occurring during 4 weeks before the last examination, as well as in the SF-36 scores, were very modest and did not reach statistical significance.

### Cytokine and chemokine levels in serum and cerebrospinal fluid

Serum and CSF samples were available on 79 patients (13 who were receiving statins and 66 who were not) for cytokine and chemokine determinations. Demographic information on the 79 patients is provided in Table 1. There were no significant differences between patients receiving statins and those who were not in CSF or serum levels for any of the 24 cytokines or chemokines measured (Table 2).

**Table 2:** Cerebrospinal fluid and serum concentrations of cytokines/chemokines in patients in the early meningoencephalitic phase of tick-borne encephalitis who were receiving and who were not receiving statins.

Cytokine/Chemokine	CSF concentrations (pg/mL) median (IQR)		P*value	Serum concentrations (pg/mL) median (IQR)		P*value
	Receiving Statins			Receiving Statins		
	Yes	No		Yes	No	
GM-CSF	2.05 (1.59–2.92)	2.05 (1.33–2.45)	0.70	0.94 (0.47–1.68)	1.01 (0.47–1.33)	0.79
IFNα	29.85 (8.33–44.76)	26.03 (18.33–39.25)	0.68	3.58 (0.30–10.69)	3.58 (0.71–7.02)	0.98
IFNγ	28.87 (9.00–70.75)	21.28 (11.85–31.24)	0.48	1.48 (0.45–2.92)	0.67 (0.45–1.19)	0.33
IL-10	0.23 (0.23–0.23)	0.23 (0.23–0.23)	0.37	0.23 (0.23–0.23)	0.23 (0.23–0.23)	0.45
IL-12P40	35.54 (9.16–58.97)	38.80 (24.01–44.31)	0.74	1.18 (1.18–1.18)	1.18 (1.18–1.18)	0.54
IL-12P70	0.72 (0.38–0.98)	0.38 (0.38–0.92)	0.51	0.38 (0.33–0.85)	0.38 (0.33–1.62)	0.77
IL-1β	1.04 (0.74–1.70)	1.17 (1.00–1.34)	0.87	0.53 (0.28–0.86)	0.41 (0.27–0.69)	0.54
IL-6	178.50 (90.15–340.80)	145.20 (111.80–247.50)	0.65	0.30 (0.30–2.70)	0.30 (0.30–0.44)	0.86
IL-8	86.01 (49.56–119.30)	69.73 (48.55–110.20)	0.62	11.08 (0.92–44.57)	2.97 (1.80–13.21)	0.65
CXCL10	7090 (2718–10960)	7563 (3467–8441)	0.87	83.60 (57.21–173.20)	101.00 (50.16–133.00)	0.94
CCL2	330.20 (216.90–617.40)	387.50 (266.40–435.00)	0.85	116.00 (85.86–189.30)	126.80 (115.60–140.20)	0.67
CCL3	11.86 (4.25–15.65)	7.55 (2.97–14.37)	0.67	1.01 (1.01–40.90)	1.01 (1.01–19.06)	0.57
TNAα	3.66 (2.07–6.04)	3.02 (1.42–6.50)	0.63	12.75 (5.91–39.05)	8.21 (3.27–17.36)	0.19
CXCL9	37.34 (10.58–67.55)	21.01 (12.98–48.20)	0.74	14.16 (8.66–28.66)	20.11 (17.72–20.71)	0.30
CCL19	137.20 (64.16–232.50)	146.20 (124.40–229.00)	0.42	11.34 (5.74–20.70)	17.09 (6.37–22.09)	0.30
CXCL13	26.24 (9.61–39.64)	22.40 (19.40–36.08)	0.46	22.88 (16.30–31.73)	23.37 (15.24–29.93)	0.89
CXCL12	7.62 (0.34–30.46)	1.93 (1.21–9.71)	0.51	36.14 (24.15–49.30)	61.32 (40.38–92.71)	0.08
IL-17F	320 (320–320)	320 (320–320)	**	320 (320–320)	320 (320–320)	0.62
IL-17A	0.31 (0.31–0.31)	0.31 (0.31–0.31)	**	0.31 (0.31–0.31)	0.31 (0.31–0.31)	0.96
IL-22	0.21 (0.21–0.21)	0.21 (0.21–0.21)	0.54	0.21 (0.21–0.45)	0.22 (0.21–0.45)	0.48
IL-21	2.71 (2.71–2.71)	2.71 (2.71–2.71)	0.68	4.75 (2.71–13.77)	7.59 (2.89–14.11)	0.53
IL-23	250 (250–250)	250 (250–250)	**	250 (250–250)	250 (250–250)	0.92
IL-25	320 (320–320)	320 (320–320)	**	320 (320–320)	320 (320–320)	0.13
IL-27	278 0 (1180–6170)	3990 (2230–4840)	0.58	2660 (1690–3870)	2490 (1850–3390)	0.70

* Mann-Whitney test was used for the comparisons.

** Not calculated due to the absence in variability (all the values were at or below the limit of detection and adjusted to 320 or 250 pg/mL).

## Discussion

Because of their widespread use as a cholesterol lowering drug, coupled with their anti-inflammatory and anti-microbial properties, statins have gained increasing interest in having a potentially beneficial role in infectious disease. The attractive concept is that statins could reduce inflammation-induced tissue pathology without altering the risk or worsening of the infection. However, this topic remains highly controversial.

Our study evaluated pre-existing use of statins in a large and very-well defined cohort of patients with TBE. We could not prove that pre-existing use of statins affected either the severity of the acute illness or the long-term outcome of TBE. Using univariate and multivariate logistic regression, we did not find an association between statin usage and the absence of severe illness defined clinically by the presence of meningoencephalitis or meningoencephalomyelitis. When severity of the acute illness was classified by a severity score using a univariate linear regression model, the use of statins was associated with having a more severe illness—patients receiving statins had on average a 3.1 point greater severity score (95% CI: 0.6 to 5.6) than those who did not receive statins. However, this association was lost in the multivariable model, where the estimated effect was close to 0. These observations can probably be explained by the strong association observed between the use of statins and certain other covariates, predominantly older age and the presence of underlying illnesses.

Receiving statins was also not associated with a favorable outcome defined by the absence of PES at 6 months, 12 months or 2–7 years after the acute illness. Moreover, patients receiving statins had a significantly higher frequency of fatigue, myalgia/arthralgia and memory disturbances within 4 weeks before the final evaluation compared with those who were not treated with statins. They also scored significantly lower in the SF-36 physical functioning and general health perceptions domains, as well as in the global overall physical component. However, after correction for age, these differences were not statistically significant, suggesting that age, rather than statin use, had the predominant effect in these findings.

Although an association between statin usage and a milder course or a better outcome of TBE was not found in the present study, the possibility of having milder disease from preexisting statin use could not be excluded due to the large CIs, presumably due to the relatively small sample size. Assessment of the severity of acute illness based on a simple clinical definition was performed for 700 patients, assessment based on the symptom severity score for 460 patients, and assessment of the long term outcome 2–7 years after acute illness for 410 patients. However, the absence of a statistically significant difference in the levels of cytokines/chemokines in serum or CSF between patients with TBE receiving and not receiving statins additionally suggests that pre-existing statin usage does not influence the course of TBE caused by the European virus subtype. Additional limitations of the present study are that the effect of statins on TBE caused by Siberian and Far-Eastern virus subtypes was not investigated and that the findings are limited to an adult population and may not pertain to children.

## Conclusions

In the present study we were not able to demonstrate a favorable impact of statins on the severity of the acute illness or on the long-term outcome of TBE. Overall, our study adds to the knowledge base on the impact of statins on various infections. Some studies have shown benefit [10–12, 15], while others have not [8, 13, 14, 16, 18, 25]. Our study provides compelling evidence that studies evaluating the risk-to-benefit ratio of pre-existing statin treatment on the outcome of infections must control for other variables associated with taking statins that could significantly affect outcome.
